# Many Cells Make Life Work—Multicellularity in Stem Cell-Based Cardiac Disease Modelling

**DOI:** 10.3390/ijms19113361

**Published:** 2018-10-27

**Authors:** Brian X. Wang, Worrapong Kit-Anan, Cesare M. N. Terracciano

**Affiliations:** 1 National Heart and Lung Institute, Imperial Centre for Translational and Experimental Medicine, Imperial College London, London W12 0NN, UK; brian.wang15@imperial.ac.uk; 2 Department of Materials, Department of Bioengineering and Institute of Biomedical Engineering, Imperial College London, London SW7 2AZ, UK; worrapong.kit-anan12@imperial.ac.uk

**Keywords:** disease modelling, patient-specific, human induced pluripotent stem cells, cardiomyocyte, personalized medicine, microenvironment, hereditary diseases, drug screening, non-myocyte

## Abstract

Cardiac disease causes 33% of deaths worldwide but our knowledge of disease progression is still very limited. In vitro models utilising and combining multiple, differentiated cell types have been used to recapitulate the range of myocardial microenvironments in an effort to delineate the mechanical, humoral, and electrical interactions that modulate the cardiac contractile function in health and the pathogenesis of human disease. However, due to limitations in isolating these cell types and changes in their structure and function in vitro, the field is now focused on the development and use of stem cell-derived cell types, most notably, human-induced pluripotent stem cell-derived CMs (hiPSC-CMs), in modelling the CM function in health and patient-specific diseases, allowing us to build on the findings from studies using animal and adult human CMs. It is becoming increasingly appreciated that communications between cardiomyocytes (CMs), the contractile cell of the heart, and the non-myocyte components of the heart not only regulate cardiac development and maintenance of health and adult CM functions, including the contractile state, but they also regulate remodelling in diseases, which may cause the chronic impairment of the contractile function of the myocardium, ultimately leading to heart failure. Within the myocardium, each CM is surrounded by an intricate network of cell types including endothelial cells, fibroblasts, vascular smooth muscle cells, sympathetic neurons, and resident macrophages, and the extracellular matrix (ECM), forming complex interactions, and models utilizing hiPSC-derived cell types offer a great opportunity to investigate these interactions further. In this review, we outline the historical and current state of disease modelling, focusing on the major milestones in the development of stem cell-derived cell types, and how this technology has contributed to our knowledge about the interactions between CMs and key non-myocyte components of the heart in health and disease, in particular, heart failure. Understanding where we stand in the field will be critical for stem cell-based applications, including the modelling of diseases that have complex multicellular dysfunctions.

## 1. Introduction

Heart failure is a global pandemic affecting over 26 million people worldwide and is becoming increasingly prevalent with an ageing population [1]. Despite the significant advances in therapies and prevention, mortality and morbidity are still high, and quality of life is poor. Current treatments delay the progression of the disease, but there are still no treatments to effectively reverse the maladaptive changes that occur in remodelling. Earlier identification of patients with a predisposition to the disease due to genetic or environmental factors or understanding key therapeutic targets in the disease progression would allow both earlier prevention and more effective treatments to be developed. 

Despite our increasing knowledge about factors influencing the initiation and progression of heart failure, historical and current study designs are unable to map the intricate interactions between cardiomyocytes (CMs) and their surrounding environment in an accurate model of the disease. Major limitations when modelling heart failure include species mismatch when using CMs isolated from animals [2], in vitro human CM models lacking the native extracellular interactions with non-myocyte that modulate CM phenotype [3], and lack of patient specificity in modelling this complex condition [4]. The recent advancements in induced pluripotent stem cell (hiPSC)-derived cell types have broadened an avenue for the development of more accurate in vitro disease models. However, more needs to be done in understanding the native cell-cell and cell-matrix interactions to fully realize the potential of hiPSCs.

In this review, we discuss the disease models of the physiological and pathological composition of the myocardium, paying a particular focus on the potential that stem-cell derived cell types present in developing an accurate in vitro model of heart failure, and also to the key myocyte-non-myocyte interactions that have been delineated thus far—these findings must be considered in future models. 

## 2. Heart Disease Models

There is clear clinical relevance in being able to accurately model human cardiac diseases in vitro. The withdrawal of drugs from the market due to unobserved toxic effects is unfortunately common. A systematic review identified that in the US, 14% of post-marketing drug withdrawals between 1953 and 2014 occurred due to cardiac toxicity [5]. Up until today, virtually all models of disease modelling and drug screening heavily rely on the use of CMs from animal models, or isolated CMs as a single cell type [6,7]. Historically, these have been grown in 2D and/or 3D cultures in an artificial environment under chemical, mechanical and electrical stimulation very different from the native environment. Among the challenges of these models are the high costs, difficult manipulations, ethics, and poor predictive capacity. Furthermore, these in vitro systems, although informative, cannot closely recapitulate the dynamics of the biological and mechanical properties of the complex, native, 3D environment, hence, lacking physiological and disease features. We summarize the benefits and limitations of currently available disease models in Table 1. These limitations must be overcome if we are to optimize a model for human cardiac failure.

### Stem Cells

In our search for an accurate disease model for human cardiac failure, a huge asset has been the development of human stem cell-derived cells. Human embryonic stem cells were first isolated from human embryos in 1998 [27,28,29]. The major limitation to their use is that the phenotype of the human from the source of the embryo is unknown. They only carry a good predictive value when the embryo is known to carry a specific highly penetrant disease mutation. Importantly, this process also involves ethical issues surrounding cell sourcing.

A new approach for producing stem cell-derived cells is through human induced pluripotent stem cells (hiPSCs) [28,30,31]. Unlike other cells, hiPSCs reflect a person’s unique genotype because they are derived from a patient’s somatic cells. hiPSCs exhibit properties that render them uniquely qualified as model systems for studying human diseases: they are of human origin, so they carry human genomes; they are pluripotent, so they can be differentiated into any of the human body’s somatic cell types; and as stem cells, they can be an autologous source of cells for medical application. Importantly, the patient specificity of hiPSCs offers the opportunity to study cells genetically matched to individual patients, and together with genome-editing tools, they allow us to introduce or correct genetic variants.

**I**nitial progress has been made in using hiPSCs to better understand cardiomyopathies [32], rhythm disorders [33], valvular and vascular disorders [34], and metabolic risk factors for ischemic heart disease [35]. In the last decade, CMs have been extensively derived from hiPSCs [36,37]. In disease modelling, many researchers have reprogrammed patient-specific cells or performed genetic editing to replicate diseases, as shown in Table 2. Various human pathologies, including long-QT syndrome (LQTS), Brugada syndrome, Timothy syndrome (also called LQT8), and catecholaminergic polymorphic ventricular tachycardia (CPVT) have been modelled with hiPSC-CMs; all faithfully recapitulating the cardiac phenotypes observed in patients [38].

However, despite the benefits that hiPSCs have over other sources of CMs in disease modelling, it is important to note the immaturity in structure and electrophysiology of hiPSC-CMs, reviewed extensively by our group [60]. These, in vitro, have a phenotype akin to neonatal human CMs, and exhibit a relatively small size, a reduced electrical excitability, inefficient excitation-contraction coupling mechanisms, and an incomplete adrenergic response, as shown in Table 3. Indeed, investigators modelling arrhythmogenic right ventricular dysplasia using hiPSC-CMs described how it was essential to induce more mature metabolic properties to accurately model adult-onset cardiac disease [35]. The immaturity of hiPSC-CMs in culture is thought to be due to, at least in part, a lack of key extracellular mediators of CM maturation. During the development of the human heart, the myocardium undergoes a complex series of structural changes that terminates in the formation of the health, adult phenotype. The maturation of CMs in vivo is regulated by a diverse library of factors, including topographical, mechanical, biochemical, electrical, and cellular interaction cues. Understanding how these cues mediate the maturation of CMs can also help us unlock the mechanisms that maintain the healthy phenotype and mediate changes in diseases such as heart failure. 

## 3. The Composition of the Healthy Myocardium

The CMs are the contractile cells of the myocardium, but it is important to consider the adult human heart as a multicellular organism. The healthy adult human myocardium is composed of many cell types, with the most abundant cell types thought to be CMs, fibroblasts, endothelial cells, and perivascular cells. CMs occupy most of the myocardium volume; between 70–80% in the adult and constitutes 30–40% of the cells by number [95]. Although estimates for the density of non-myocyte components (fibroblasts, endothelial cells, smooth vascular muscle cells, lymphocytes and neurons) of the heart vary substantially, they are widely agreed to be vital for the normal homeostasis in health; non-myocyte cell types and the matrix provide the chemical, electrical, and mechanical stimulation for the CMs, and form the structures essential for the vascular supply required for efficient CM contraction, optimal shape and functions and long-term survival. This has been shown in numerous studies in which the isolation of human CMs leads to a rapid loss of contractile function, and prominent changes in the structure [17,18,19,20]. Grossly, there is a loss of the rod-like morphology and changes in the ultrastructure domain of the excitation-contraction coupling complexes; most notably, there is a detubulation–a loss of the transverse-tubules required for rapid, synchronous calcium-induced calcium release (CICR). Together with the change in ECM composition in the development of the myocardium, it is clear that the extracellular interactions of the CMs are vital in the maintenance of the healthy adult myocardium, and the interactions, at least in part, drive the changes that occur in diseases. 

### 3.1. Heart Disease Remodelling and Its Consequences

Cardiac remodelling is defined as a group of molecular, cellular and interstitial changes that manifest clinically as changes in size, mass, geometry, and function of the heart after stimulation and stimuli at disease onset such as cardiac ischemia, inflammation, pressure overload, and pharmacological toxicity [96,97,98,99]. The main consequence of cardiac remodelling is contractile dysfunction, leading to left-sided heart failure [100]. There are two major types of left-sided heart failure; heart failure with a reduced ejection fraction, also called systolic failure and heart failure with a preserved ejection fraction, also called diastolic failure. Common to both is left ventricular myocardial remodelling, and progressive loss of ventricular function, asymptomatic at first, but evolves to signs and symptoms of heart failure. This results in a poor prognosis due to its association with ventricular dysfunction and malignant arrhythmias [101,102]. Remodelling involves various mechanisms associated with the pathophysiological role of different factors, including cell death, energy metabolism, oxidative stress, inflammation, extracellular matrix protein, contractile proteins, calcium transport, geometry, and the neurohormonal activation of CMs and non-myocytes [71,103,104]. Under the biomechanical overload seen in diseases, CMs and non-myocytes respond to the circulating neurohormones and cytokines in the altered environment via multiple mechanisms including the integrin-extracellular matrix interactions and renin-angiotensin-aldosterone axis activation as well as through the release of myocardial hormones and cytokines. The key non-myocytes components participate in these changes includes fibroblasts [105], endothelial cells [106,107], lymphocytes [108], and neurons [109,110]. They undergo, for example, a transition of cardiac fibroblasts into the more active myofibroblasts, resulting in an accumulation of extracellular matrix proteins [111], a shift in the actions of the endothelium toward reduced vasodilation [112,113], a proinflammatory state, and prothrombic properties and recruitment of immune cells [114] amongst others. This remodelling leads to, at a macroscopic level, an increased deposition and the alteration of the cardiac ECM, and subcellularly leads to CM hypertrophy, dysfunction, and death. These maladaptive changes participate in the pathogenesis of cardiac dysfunction.

### 3.2. Multicellularity

As we previously described, CMs and non-myocyte intercellular interactions are central in the initiation and progression of cardiac dysfunction. Therefore, CM-specific analyses cannot model all cardiac diseases. Future models should use various cell types including CMs, fibroblasts, endothelial cells, vascular smooth muscle cells, lymphocytes, and neurons and the ECM (Table 4), as well as considering the cells recruited in disease. Despite the immaturity of stem cell-derived differentiated cell types, investigating how the changes occur in remodelling contributes to the pathogenesis of heart failure using models that utilize these cells, especially hiPSC-CMs, and offers huge potential in mapping this complex disease.

#### 3.2.1. Extracellular Matrix (ECM)

The complex tissue and organ architecture of the heart is maintained by extensive ECM networks, composed of fibrous proteins (e.g., collagen, elastin), adhesive glycoproteins (e.g., fibronectin, laminin), and proteoglycans. These guide the anisotropic alignment of CMs, form the mechanical environment for cells and contribute to the stress-strain relationships of the heart. In health, the fibres of the ECM use the energy produced in systole for the re-lengthening of CMs during diastole. It is important to note that in heart failure there is a CM hypertrophy as well as collagen deposition in the ECM [104,111,117]. Though hypertrophy is common in many adults, in particular, trained athletes, the changes in the ECM composition is key to differentiating pathological from physiological hypertrophy. The increase in collagen deposition in the ECM during cardiac remodelling, despite preserving an adequate cardiac output in the early phases of heart failure, proves to be chronically maladaptive in contributing to ventricular dysfunction and conduction abnormalities. It is widely agreed that the laying down of new ECM proteins in remodelling is regulated at a cellular level by a plethora of molecular, mechanical, and hemodynamic factors. Many researchers have studied the role of ECM on in vitro cultures of hiPSC-derived cells. For example, 3D cardiac cultures on different ECM matrices directed hiPSC-CMs to different cell fates [104,111,117]. Delineating the mediators of this would go some way to providing insight into therapeutic targets and more accurate models of human cardiac disease. 

#### 3.2.2. Fibroblasts 

Cardiac fibroblasts account for 20% of the non-myocyte components of the heart [132]. The numbers fluctuate as the heart develops but they are substantially stimulated postnatally as the neonatal heart begins to function independently, likely correlated with the need for a greater mechanical and tensile strength as the nascent myocardium. In addition to their mechanical properties, they produce various proteins found in the ECM, including the major fibrillary collagens type I and III, which comprise the bulk of the ECM, as well as collagenases, fibronectin, and vitronectin [133]. The proliferative capacity of cardiac fibroblasts and the differentiation into the more active disease phenotype, cardiac myofibroblasts, has allowed us to investigate the effects these cells have on CMs in culture. Our group recently showed, using co-culture setups with hiPSC-CMs and human cardiac fibroblasts, that although distant paracrine interactions independent of contact between the two cell types causes the prolongation of hiPSC-CM calcium transients, the direct seeding of fibroblasts on CM monolayers improves the efficiency of CICR and a contribution of the sarcoplasmic reticulum to decay mechanisms, closer recapitulating the parameters of the adult healthy human CM [134]. Future studies must identify the significant mediators of this modulation.

#### 3.2.3. Endothelial Cells

The vasculature is critical in the delivery of oxygen and nutrients to CMs and, thus, momentum has gathered in the modelling of vascularized microenvironments [113,135]. In the context of disease, the vascular endothelium is critical in the initiation of the inflammatory response, the triggering of inflammation, the regulation of vasomotor tone, and the control of the vascular permeability [112]. The importance of endothelial cells has been shown in fibrin gel co-culture constructs, where endothelial cells and patient-derived pericytes increase the stability of perfusable micro-vessels [25]. The benefit of these microfluidic models is that it requires comparably small amounts of cells and reagents, as well as being much easier for the constant monitoring and manipulation of the construct configuration, compared to large artificial tissue studies. Endothelial dysfunction plays a significant role in the development of atherosclerosis and the consequential strokes or myocardial infarctions. hiPSC-derived endothelial cells possess a repertoire of phenotypic plasticity and are amenable to cell-based assays probing endothelial contributions to inflammatory and cardiovascular diseases [136,137]. Folkman et al. produced de novo growth of capillary tubes in vitro. A key factor identified was Vascular Endothelial Growth Factor, but it is clear that multiple factors and cell types are required to recreate the full myocardial vascularity in 3D models. The lack of evidence for functional vessel formation in models utilizing hiPSC-endothelial cells hinders investigations into the role that these cells play in the development and maintenance of the adult myocardial phenotype [138,139]. It is postulated that this is due to the immaturity in these iPSC-derived cells, similar to hiPSC-CMs, and thus, an improvement in hiPSC-endothelial cell maturation is needed before we can obtain evidence of functional vessel formation in human models of cardiac disease. 

#### 3.2.4. Vascular Smooth Muscle Cells

SMCs display plasticity in switching expression between contractile and proliferative (synthetic) phenotypes in the regulation of vessel tone and blood pressure. The challenges to studying diseases of the vasculature include the limited proliferation capacity and rapid senescence of adult human SMCs and hiPSC-SMC. Many studies have shown that the in vitro modulation of hiPSCs can direct them to become specific subtypes of SMCs; displaying a proliferative, synthetic or contractile phenotype. These SMCs closely resemble native SMCs at both the transcriptional and functional levels, with their pluripotency effectively silenced. There is promising evidence to suggest that SMCs have a significant role in the construction of the tunica media in the maintenance of vessel tension and contraction-relaxation capabilities and can be reproducibly created using SMC. However, less reliable is the evidence for the formation of functional vessels using hiPSC-ECs and SMCs in the co-culture. It is believed that the pattern of gene expression varies between in vivo and in vitro studies, as well as in the somatic tissue from which the stem cells are sourced [140].

#### 3.2.5. Lymphocytes

The human body’s adaptive immune system is designed to protect the body from injury. The central cellular components of this immunity are T- and B-cells that arise from lymphoid progenitor cells in the bone marrow. The cellular components of innate immunity are myeloid cells, including monocytes, macrophages, dendritic cells, natural killer cells, as well as neutrophilic, basophilic, and eosinophilic granulocytes. Heart failure is a state of chronic inflammation [97], with a characteristic increase in circulating and myocardial pro-inflammatory cytokines that have been shown to promote pathological left ventricular remodelling. Though pre-clinical and early human studies suggest a therapeutic role for cytokine antagonism in heart failure, the poor characterization of the cascade of events that occur in the inflammation in heart failure is thought to be key in the failure of immunomodulation trials. 

Studies in which coronary ligation in adult C57BL/6 mice has been used to mimic chronic ischemic heart failure showed that the initial injury causes a global expansion and activation of CD4+ T-lymphocytes and the expansion of memory T-cells in the spleen. The key findings are that the cardiac and splenic T-cells in heart failure are primed to induce cardiac injury and remodelling (long-term left ventricular dysfunction, fibrosis, and hypertrophy), and retain this memory upon the adoptive transfer from donor mice to naïve recipient mice [141]. There is extensive evidence to show that CD4+ T-cells contribute to the size of the infarct following a myocardial ischemia-reperfusion injury. Recently, regulatory T-cells were shown to contribute to the rosuvastatin-induced cardioprotection against myocardial-reperfusion injuries [142]. The degree of recruitment, the activity of neutrophils, and the most numerous leukocyte subset in the first hours after ischemia-reperfusion critically influence the extent of the injury. Though there are limited studies into the roles of other lymphocytes in this disease and much of the evidence is from mouse myocardial infarction models, the importance of considering the lymphocytes in their individual subsets when identifying their role in the disease and as therapeutic targets is clear [128].

#### 3.2.6. Neurons

The dynamic interconnections between the heart and the neuronal system that controls cardiac function are bidirectional; dysfunction in either system can trigger a cascade that alters the functioning of the other. Peripheral neural networks are composed of all the necessary neural machinery for the reflex control of the heart [124]. The integrated, autonomic nervous system that has tonic control over cardiac function in the quiescent environment can efficiently respond to stressors by altering the work done by the myocardium. Crosstalk between neurons and CMs in vitro has been shown to improve the functioning of both [143]. The link between the two cell types is well documented in hyperglycemia-related neuropathy secondary to diabetes, whereby the risk of developing cardiac disease is higher than in non-diabetic patients [125,126]. Conversely, neuronal dysfunction has also been seen to follow cardiac injury; in the case of scar formation in cardiac ischemic patients, it causes a delay in the conduction velocity and/or inducing cardiac arrhythmia, which leads to contractile dysfunction and, eventually, heart failure [127]. It is therefore clear that there is a close interplay between the two cell types, and future studies must consider the temporal relationship between events that conclude with chronic heart disease. 

Current disease modelling simplifies the complexity of the heart by using a simple co-culture; whereas cardiac organoids have not yet been reported [144]. Recently, Ronaldson-Bouchard et al. demonstrated a structural improvement of hiPSC-CMs co-culture with primary human fibroblasts and endothelial cells. Although this 3D cardiac tissue more closely mimics the physiological environment of the heart, a co-culture with primary cells from different sources compromises their patient specificity. Therefore, these models cannot reach the potential that we envisage of multicellular models based on autologous hiPSC cell types in the future. 

Looking ahead, beyond modelling the heart as a closed system, we must also consider its place as an organ within the human circulatory system. A number of clinical and animal studies have implicated inflammation as a key contributor to myocardial remodelling. Prolonged exposure to inflammatory cytokines not only triggers signalling cascades within the heart but also other organs [145,146]. The importance of the cardio-splenic axis has been shown in animal studies, in which mice without a spleen are shown to have an attenuation of lymphocyte production and reduced cardiac dysfunction [147]. The importance of the kidneys has been shown clinically, whereby patients with chronic kidney diseases experience higher rates of mortality following myocardial infarction [148,149,150]. It is therefore critical that signalling from beyond the myocardial cell types must be considered in order to develop a model that fully recapitulates the heart in disease. 

## 4. Conclusions and Future Prospects

Our knowledge of human diseases has improved enormously over the last few decades, owing to advancements in the in vitro and animal models that allow us to investigate the intercellular interactions between CMs and non-CM cell types, demonstrating their importance in simple 2D co-cultures, as well as within a 3D scaffold. Increasingly, it has become standard to use hiPSCs, especially hiPSC-CMs, in models for human diseases due to their patient’s origin. However, with many of the models of human diseases dependent on the ability of the CMs to recapitulate native adult human CM physiology, differences between CMs in vitro and in the native myocardium pose challenges to fully realizing the potential of human disease models. The advancements in stem cell biology and tissue engineering have spearheaded the development of in vitro cardiac models that can employ patient- or disease-specific CMs and non-myocytes in culture. As it becomes increasingly clear that CMs are intricately modulated by the extensive extracellular interactions in the native myocardium, more accurate future in vitro myocardial models will be more physiologically relevant with the ability to provide multiple biochemical, mechanical and electrical readouts, for real-time monitoring of disease progression and functional endpoints. The development on new biomaterials will not only provide support to patient-specific stem cell-derived cells but will also contribute to their maturation and their guiding alignment, providing the relevant stiffness and be dynamically replaced over time by ECM components secreted from the cells [31,151,152,153,154,155]. Additionally, the multidisciplinary approach that integrates in vitro, in vivo, and ex vivo datasets by using in silico computational methodology for analysis and prediction could potentially generate new insights in Cardiology [156]. 

Overall, a more solid picture of the myocyte-non-myocyte interactions that occur in the native myocardium using relevant models will enable us to develop a greater understanding of how the adult myocardium functions. This will provide the unique opportunity to study strategies for disease intervention in human in vitro disease models, spanning the gap between 2D culture and in vivo testing, thus reducing the cost, time, and ethical burden of the current approaches.

## Figures and Tables

**Table 1:** The summary of the benefits and limitations of currently available disease models.

Model Type	Description	Benefits	Limitations	Ref.
Animal	Animals with defined genetic background or subject to acute intervention (e.g., coronary obstruction) to mimic discrete time points.	Small animals: delineate molecular pathways in early- or late-stage heart failure, aiding the identification of biomarkers and therapeutic targets. Large animals: preclinical proof of concept for novel therapies before clinical trials.	Gene expression-silencing or drug-induced pathogenesis does not recapitulate the disease initiation in humans. Many human diseases are human-specific. Differences in physiology (circulation, etc.), cardiac output requirements, myocardial composition (vascular supply, etc.), biochemical absorption, distribution, metabolism and immunoresponses.	[8,9,10,11,12,13,14]
Human-specific expression in Chinese hamster ovary (CHO) and human embryonic kidney (HEK) cells	Model protein force expression, e.g., tests the off-target effects to ion channels prolonging the QT interval.	Expression of human ion channels. Avoids the expense of whole animal studies. Reproducibility in cryogenically freezing and thawing cell lines for the stable expression of the desired channels.	Single ion channel does not recapitulate diseases in humans. Does not negate species mismatch.	[15,16]
Adult human cardiomyocytes	Isolated from diseased or non-diseased patients during surgery.	Human genome so we can map the response in humans to cardiac disease.	Limited quantities (e.g., ethical limitations). Large variability in phenotype and rapid dedifferentiation.	[17,18,19,20,21]
Organoid	3D in vitro culture systems derived from self-organizing stem cells and extracellular matrix (ECM) proteins secreted from the cells.	Higher complexity compared to the 2D models, with more extensive cell-ECM interactions and possible vessel formation.	Expensive and technically challenging setup, resulting in poor reproducibility.	[22,23]
3D cardiac tissue	3D in vitro culture systems with natural and/or synthetic ECM structural support.	Ability to manipulate ECM components enables a greater control of the scaffold composition and more complex cell-ECM interactions. Decellularized scaffold for cell adhesion mimics the naturally occurring macro and microstructures.	Limited information on cost, reproducibility, and performance.	[24,25,26]

**Table 2:** The summary of the available human induced pluripotent stem cell (hiPSC)-derived cells used in-disease modelling.

Pathology	Cell Type Involved	Mutation	(Drug/Treatment) Test	Ref.
**Endothelial**				
Healthy	EC	N/A	Flow-induced disease and simvastatin	[39]
Hutchison-Gilford Progeria Syndrome	EC	Patient-derived	N/A	[40]
**Smooth muscle cells**				
Supravalvular aortic stenosis	SMC	Elastin (ELN)	Elastin recombinant protein	[41]
Marfan syndrome	SMC	FBN1	Gene editing and drugs	[42]
**Lymphocytes**				
Healthy	B-cell lymphoid lineage	N/A	N/A	[43]
**Red Blood cell (RBC)**				
Healthy	CM and RBC	N/A	Toxicity of RBC	[44]
**CM**				
Hypoplastic left heart syndrome	CM	Patient-derived (GM12601)	Isoproterenol	[45]
Arrhythmogenic right ventricular dysplasia	CM	Plakoglobin, plakophilin-2	Metabolism induced onset	[46]
Familial hypertrophic cardiomyopathy	CM	MYH7 Arg663His	Verapamil, Diltiazem, Mexiletine among 15 drugs	[47]
LEOPARD syndrome	CM and all three germ layers	PTPN11	N/A	[48]
Friedreich’s ataxia	Neurons and CM	GAA triplet repeat expansion within the first intron of the frataxin gene	N/A	[49]
Catecholaminergic polymorphic ventricular tachycardia type 1	CM	Ryanodine Receptor 2 (RYR2)	Isoproterenol	[50,51]
LQT1,2,3,5,8,14	CM	Patient-derived	Common drugs	[52,53,54,55,56,57]
Barth syndrome	CM	Tafazzin (TAZ)	Genetic rescue	[58]
Ischemic heart damage	CM	Aldehyde dehydrogenase 2 (ALDH-2) deficiency	siRNA knockdown	[59]
Brugada syndrome	CM	SCN5A-1795insD mutation	N/A	[33]

SMC = Vascular smooth muscle cell, CM = cardiomyocyte, EC = Endothelial, RBC = Red blood cell.

**Table 3:** The summary of the differences between hiPSC-cardiomyocytes (CMs) and contractile CMs. Data extracted from References [58,60,61,62,63,64,65,66,67,68,69,70,71,72,73,74,75,76,77,78,79,80,81,82,83,84,85,86,87,88,89,90,91,92,93,94].

**Structure**				
		**hiPSC-CM**	**Atrial**	**Ventricular**
**Shape**		Any, not defined	Cylindrical	Cylindrical and bifurcated
**Volume**		Small	Large	Very large
**Sarcomere Organization**		Random	Orderly and aligned	Orderly and aligned
**Mitochondria population**		Few	Abundant	Abundant
**T-tubule organization**		Absent	Scarce	Abundant
**Glucose Metabolism**		High	Low	Low
**Nucleus morphology**		Mono	Mono, bi, multi	Mono, bi, multi
**Electrophysiology**				
**Spontaneous activity**		Very frequent	Absent	Absent
**Maximum diastolic potential**		−60 mV	−70 mV	−80 mV
**Maximum upstroke velocity**		44–187 V/s	200 V/s	200 V/s
**Action potential amplitude**		94–113 mV	80–130 mV	100 mV
* Action potential duration at 50%		60–400 ms	200 ms	200–300 ms
* Action potential duration at 90%		80–500 ms	200–400 ms	250–400 ms
**Force Generation**		100–150 Pa for a single cell	Myocardium tensile force ≈ 56 kPa	Myocardium tensile force ≈ 56 kPa
**Elastic modulus**		466 Pa	22–55 kPa	22–55 kPa
**Molecular Marker**				
**Gap junction**	Cx40	+	+	-
	Cx43	+	+	+
	Cx45	+	-	-
**Ion channel**	KCNA5	+	+	-
	NCX1	+	+	+
	SERCA2a	+	+	+
	RYR2	+	+	+
	Ca_v_ 1.2	+	+	+
	K_ir_ 2.1	+	+	+
	K_v_ 4.3	+	+	+
	KChip 2	+	+	+
	KCNH2 (HERG)	+	+	+
**Structural protein**	TNNT2	+	+	+
	ACTN2	+	+	+
	MLC2A	+	+	+
	MLC2V	+	-	+
	MYL2	+	+	+
	MYH6	+	+	+
**Master gene**	NKX2.5	+	±	±

* Action potential duration for hiPSC-CMs depends on seeding conditions and differentiation protocol.

**Table 4:** The summary of the role of non-myocyte cell types in health and the disease.

Cell.	Healthy	Disease	Notes
**Fibroblasts**	● ECM turnover, maintaining a balance between the synthesis and degradation of the matrix	● Scar formation (fibrosis) ● Increase ECM protein ● Phagocytose apoptotic cells ● Crosstalk to EC and macrophage for angiogenesis and matrix synthesis	[105,111,115,116]
**ECM**	● Periostin, laminin, vimentin, fibronectin, and collagen types I (90%), III, V, and VI ● Alignment ● Mechanical support	● Increase in collagen I, III, IV, V, and VI ● laminin, fibronectin, thrombospondin, and tenascin	[104,111,117,118,119]
**Endothelial cells**	● Structural support ● Vasculature homeostasis ● Biochemical factors such as nitric oxide, endothelin-1, IL-6 ● Progenitor of cardiac pericytes and vascular smooth muscle cells	● Inflammation (hypertrophy, inotropy, apoptosis, mitosis) ● Neovascularization increase the density of peri-infarct vessels ● Paracrine	[112,120,121]
**SMCs**	● Mechanical support of vasculature: contractile or synthetic (proliferative) mode	● Loss of elasticity ● Reduced contractile ● Increased proliferation	[122,123]
**Neuronal cells**	● Conduction fibre and pacemaker (AV, SA, Purkinje) ● Control of rhythmic beating	● Block, slow down conduction ● Essential component for embryo development	[124,125,126,127]
**Lymphocytes**	● Few residents ● Mast cells act as inflammatory mediator storage and activating the local renin-angiotensin system ● Macrophage performs a janitorial homeostasis and facilitates electrical conduction	● Macrophage has a role in ECM turnover/cell death, scar formation, neutrophil recruitment, and vascularization support	[128,129,130,131]

## Abbreviations

2D	Two-dimensional
3D	Three-dimensional
ALDH-2	Aldehyde dehydrogenase 2
CICR	Calcium induced calcium release
CM	Cardiomyocyte
CPVT	Catecholaminergic polymorphic ventricular tachycardia
CHO	Chinese hamster ovary
EC	Endothelial cell
ECM	Extracellular matrix
ELN	Elastin
HEK	Human embryonic kidney
HiPSC	Human induced pluripotent stem cell
LQTS	Long-QT syndrome
LQT8	Timothy Syndrome
RBC	Red blood cell
SMC	Smooth muscle cell
TAZ	Tafazzin
